# Enhancing CISD2 expression to retard liver aging

**DOI:** 10.18632/aging.203929

**Published:** 2022-02-28

**Authors:** Yi-Long Huang, Zhao-Qing Shen, Ting-Fen Tsai

**Affiliations:** 1 Center for Healthy Longevity and Aging Sciences, National Yang Ming Chiao Tung University, Taipei, Taiwan; 2 Department of Life Sciences and Institute of Genome Sciences, National Yang Ming Chiao Tung University, Taipei, Taiwan; 3 Institute of Molecular and Genomic Medicine, National Health Research Institutes, Zhunan, Taiwan

**Keywords:** liver aging, Cisd2, transcriptomics, proteomics, non-alcoholic fatty liver disease, oxidative stress

**Figure 1:**
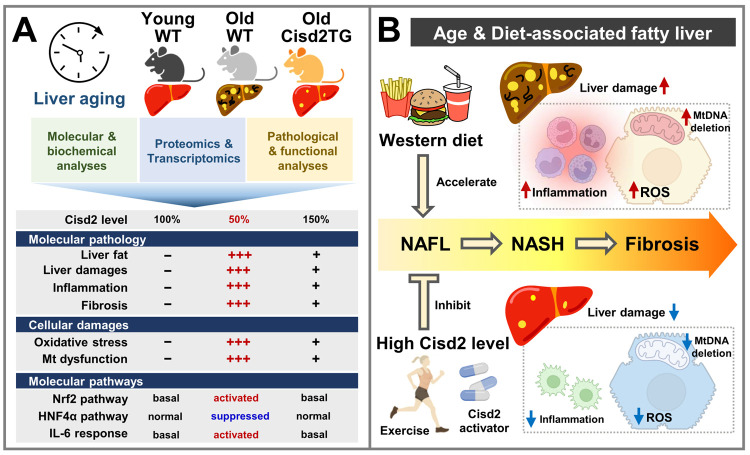
**Cisd2 attenuates liver aging and preserves a young-like proteome and transcriptome. (A)** An age-dependent decrease in Cisd2 correlates with extensive molecular, cellular and pathological alterations within the liver. Whole-body Cisd2 overexpression (Cisd2TG) attenuates aging-induced liver injury and related molecular changes, as evidenced by histopathological and 'omics analyses. **(B)** A Western diet accelerates progression of non-alcoholic fatty liver disease (NAFLD) phenotypes, namely hepatic steatosis, inflammation and fibrosis. Maintaining a high-level of Cisd2 by exercise or by the introduction of compounds that activate Cisd2 expression should protect the liver from age-related pathological changes. Figure created with BioRender.com.

Liver is a pivotal metabolic organ that is responsible for xenobiotic detoxification, protein synthesis, bile production and energetic balance. Aging of the liver manifests as multiple functional and structural alterations [1]. Cisd2, the second member of the CDGSH iron-sulfur domain-containing protein family in mammals, has been shown to serve as an anti-aging protein that can modulate longevity and maintain cellular homeostasis [2]. Here our laboratory highlights a key factor underlying liver dysfunction during aging, which is a reduction in Cisd2 expression [3]. In a series of mouse studies, the robust protective effects of Cisd2 on age-associated or diet-induced liver damage were demonstrated. In agreement with the above, a persistently high level of Cisd2 appears to slow down the aging rate of liver in mice and the results are likely to be similar in humans (summarized in Figure 1A).

On examining Western diet (WD)-related changes after 4-month treatment (at the age of 6 months), we discovered that the genetic dosage of Cisd2 negatively correlated with the severity of the non-alcoholic fatty liver disease (NAFLD) phenotype. Hepatocyte-specific Cisd2-haploinsufﬁciency (Cisd2hKO^+/−^) aggravates nonalcoholic steatohepatitis (NASH) development and upregulates expression of the mRNAs responsible for cholesterol synthesis, fibrosis and lipid metabolism [4]. Although WD-fed Cisd2 transgenic (Cisd2TG) mice show significant improvement in liver pathology compared with WD-fed WT mice, the transcriptome showed no overt changes. Thus it seems likely that there are other post-transcriptional mechanisms contributing to the resistance of Cisd2TG mice to WD challenge. RNA sequencing and proteomics analysis have provided promising results regarding aspects of natural aging [3,5]. Cisd2 overexpression blocks the significant age-dependent accumulation of liver fat and maintains a young-like transcriptome and proteome expression pattern even at an advanced age. In particular, quantitative proteomics data show that many proteasome subunits induced by aging are suppressed in Cisd2TG mice, whereas multiple chaperones that are normally suppressed by aging are induced in Cisd2TG mice [5]. The impact of Cisd2 on proteostasis is likely to be a contributing factor to longevity. Consistent with these results, age-related induction of oxidative stress and oxidative damage to proteins, lipids and mitochondrial DNA are all significantly decreased in old Cisd2TG mice compared to old WT mice. It has been proposed that overproduction of ROS leads to damage to mitochondrial DNA and any impairment of mitochondrial function then elicits further ROS production. Persistently high level of Cisd2 may attenuate this self-accelerating vicious cycle and consequently create a delay in the development and progression of age-related liver diseases. Although Cisd2 appears to have an impact on a number of aging hallmarks, including oxidative stress, proteostasis, inflammation and metabolic regulation, there remains a definite need for more research in order to understand the exact effects of Cisd2 in hepatocytes.

Based on transcriptomics data, HNF4a (Hepatocyte nuclear factor 4 alpha) has been identified as one of the most signiﬁcantly inhibited upstream regulators among various age-related genes. Most Hnf4a-related transcription changes have been found to be attenuated by Cisd2TG. Hnf4a is a key regulator of liver functions through its effects on downstream genes. One recent study found that upregulating Hnf4a expression in NAFLD helps to re-establish normal liver function [6]. Therefore, Hnf4a activation is proposed as a mechanism that will deliver the benefits of Cisd2TG. We note that in the AML12 cell model, even in the absence of a liver-specific microenvironment, Cisd2 deficiency results in lipid accumulation, mitochondrial dysfunction, and oxidative stress, as well as an induction of the Hnf4a target gene Apoa4. These findings suggest that Cisd2 functions in a cell-autonomous manner in hepatocytes and that the Hnf4a transcription network is involved in this liver-specific regulation.

Intriguingly, age-associated transcriptional signatures have been found to show few comparable changes to age-related proteomics profile changes based on our two 'omics datasets. The reason for this inconsistency is their different sensitivities and the distinct dynamic ranges of detection between RNA-sequencing transcriptomics and mass spectrometry-based proteomics. Additionally, it is possible that age affects gene expression at different levels of regulation. Therefore, integration of 'omics data at the mRNA, protein, or even metabolite level, is likely to more comprehensively reflect age-dependent or Cisd2-related alterations. Future work should try to directly assess the hepatic functions that parallel these molecular changes.

In conclusion, our findings support the idea that an enhancement of Cisd2 protein expression protects against hepatic aging. Several pathways are potentially associated with liver aging and Cisd2-mediated longevity and these were able to be identified by 'omics-based comparisons. This suggests that a restoration of hepatic Cisd2 protein expression in middle-aged or old-aged mice is likely to rejuvenate their aged livers. More importantly, whether such a treatment regimen correlates with an age window or there is a suitable time-frame during the aging process can be manipulated warrants further study. Notably, our laboratory has shown that exercise training regimens induce expression of Cisd2 [7]. Thus it would seem that small molecules elevating Cisd2 levels and/or change in lifestyle, such as modification to the diet and increased exercise, offer opportunities to treat age-related liver dysfunction (Figure 1B).
